# Psychiatry and the unknown future: the period of hope

**DOI:** 10.3389/fpsyt.2026.1824703

**Published:** 2026-06-24

**Authors:** Andreas Conca, Giancarlo Giupponi, Klaus Eisendle, Filippo Boschello, Fabian Max Wedmann

**Affiliations:** 1 Bolzano Central Hospital, Bolzano, Italy; 2 Paracelsus Medizinische Privatuniversitat, Salzburg, Austria; 3 Claudiana Scuola Provinciale Superiore di Sanita, Bolzano, Italy

**Keywords:** complex system theory, digital pheno typing, meaning, multi level network, synergetics

## Abstract

Modern psychiatry is transitioning from an entity-based taxonomy toward a systems-oriented science that integrates biological, psychological, social, and existential levels of analysis. We propose a conceptual framework that combines genetics and multi-omics approaches, synergetics, complex systems theory, and a neo-existentialist perspective. Within this framework, psychiatric illness is conceptualized as a crisis of regulation and meaning emerging across dynamically interacting multi-level networks. We discuss implications for clinical practice, including process-based psychotherapy, idiographic mapping, and neuromodulatory interventions, as well as consequences for service design, such as recovery-oriented integrated care ecosystems and ethical challenges in forensic settings. Finally, we outline key research directions, including multimodal clustering approaches and digital phenotyping, aimed at capturing non-linear trajectories of mental suffering and recovery. “In order to possess what you do not possess…” ― T.S. Eliot

## Introduction

The biopsychosocial model provides a framework for understanding the multifaceted origins of psychiatric disorders. This model emphasizes the interplay of biological, psychological, and social factors in their causation, treatment, and prevention (1). The so called mental disorders can be currently characterized by manifold perspectives. On one side genomics, proteomics, transcriptomics, metabolomics, connectomics and neuroimaging technique constantly offer new fruitful insights (2–5). On the other one inter- and intrapersonal psychodynamic theories along with complex socio behavioural and cultural influences and even spirituality play undeniably a pivotal role (6–8). At the moment we can stress the hypothesis of six dimensions as a model to explain, to define and maybe to cure psychiatric disorders (9):

1. The molecular level: we can measure and analyze big data and genotyping to understand the genetic basis of these disorders. This provides a foundational understanding of the biological factors involved.
2. The cellular level: we are able to explore and examine the regulatory circuits within cells. These circuits play a crucial role in maintaining cellular function and, when disrupted, can lead to various psychological conditions.
3. The neuro-anatomical circuits: they are observable through advanced imaging techniques. These circuits involve the intricate connections and pathways within the brain which are correlated to cognition, emotion, behavior and biorhythms.
4. The psychological level: we focus on measurable regulatory circuits that thoughts, emotions, behaviors and biorhythms. Understanding these circuits helps in identifying patterns and triggers associated with psychological disorders.
5. The level of symptoms: we can perturb these regulatory circuits to study their effects and potential therapeutic interventions. This involves experimenting with different treatments to observe changes in symptoms and overall bio-psycho-social health.
6. The social regulatory circuits: are influenced by correlations and feedback within social interactions. These circuits highlight the impact of social environment and relationships on bio-psycho-social health, emphasizing the importance of considering social factors in treatment and prevention strategies.

All these mutually self-influencing dimensions are critically involved in bio psycho-social health manifestations in the framework of a dynamic complex interplay. However valuable, the classical biopsychosocial paradigm may be considered as insufficient to capture the 1) inherent complexity and 2) non linearity which distinctively characterize psychiatric disorders (10–12). Contemporary network models of mental disorders (e.g., 11) converge on the view that psychopathology emerges from dynamic feedback loops among symptoms, behaviors, and contextual factors. While primarily operating at the symptom and psychological level, our framework extends this logic vertically across biological, social, and existential domains within a broader synergetic and anthropological perspective.

At the level of service organization, similar principles have long been implemented in recovery-oriented community psychiatry, which adopts an ecological and systemic understanding of mental illness. Community-based care emphasizes transdisciplinary teamwork, continuity, and active service-user involvement within relational and territorial contexts (13). Recovery is thereby conceived not merely as symptom reduction but as the restoration of agency, meaning, and social participation, aligning closely with network and synergetic models in which small, well-timed interventions can foster qualitative reorganization in complex systems (14, 15).

Synergetics, a theory originally developed in physics and chemistry, offers a promising avenue for improving the traditional biopsychosocial paradigm and understanding these two key features within the neuroscience field. Synergetics primarily focuses on the empirical study of systems in transformation (16). It emphasizes how systems behave as a whole, by producing emergent properties secondary to the mutual interactions of the system’s components. Properties that cannot be predicted by examining the individual components in isolation. The synergetic model may explain how the cooperation between individual parts of a system produces new structures or functions at the macroscopic level by means of a self-organization process (17, 18). By self-organization it is meant that the resulting new structure or function are not imposed on the system from the outside. The system finds them by itself by reaching a stability phase after a previous critical instability triggered by internal or external factors (12, 16, 19). Looking at the possible evolutions of the biopsychosocial paradigm at least two questions of epistemiological character inevitably arise. The first one: 1) if the domains within this paradigm are mutually connected, what kind of connections must be assumed and what could drive a change. And the second one: 2) what role does the relation brain vs mind play within this complexity?

To address the first question, it is worth mentioning that, according to the synergetic model, complex systems are composed of individual structures, whose degree of interconnectedness is chaotic and whose changing interactions lead to non-linear developments (20). Phenomena like cognition, memory and language, positive and negative emotions, social behaviour and biorhythms may be understood as emergent properties arising from an intricate interplay of interconnected components within the individual self and their interaction with the environment. By instability phases, the permanent variations regarding the system’s parameters occur (evolutionary) in ever-shorter timescales (dynamic). The system counteracts the ever-increasing risk of disorder and disorganisation (entropy) by increasing its control and protection functions (stability), which are based above all on feedback processes. The more complex the systems become, the more their feedback possibilities increase, the more likely it is that completely new system properties (emergency) can emerge (20, 21).

According to Synergetics, some systems may operate in a stable and predictable manner, while others, or even the same system under different conditions, can undergo significant transformations in response to seemingly small internal or external perturbations (22). Small perturbations may have negligible effects when a system is strongly “attracted” to a particular state. The term “attractor” refers to a stable state or a stable pattern of behaviour within the system’s components (15, 23). Conversely, when the system is near to a critical point (the “kairos” moment), even minor perturbations may trigger a shift leading to a new stable state.

The notion of kairos as a critical moment of qualitative transformation resonates with classical crisis theory, which conceptualizes periods of acute instability as both dangerous and potentially growth-promoting phases (22). Early crisis models, and more recent formulations (e.g., 24) emphasize that small interventions during such windows may disproportionately influence system reorganization. Within our framework, kairos represents a synergetic translation of crisis theory into a multilevel systems perspective.

Importantly, kairotic moments do not imply the need for simultaneous intervention across all levels of the system. Rather, they require clinical parsimony and prioritization: the clinician is called to identify the level at which instability is most immediate, irreversible, or threatening to system integrity, and to target this level as an entry point for stabilization. In this sense, interventions at one level (e.g., biological, psychological, or social) function as enabling conditions that restore sufficient stability, thereby reopening adaptive degrees of freedom for subsequent multilevel reorganization.

In this framework, the term “butterfly effect” refers to the high sensitivity of the system’s initial conditions to change. Dynamic complexity research suggests that the process of environmental adaptation is characterized by order transitions from lower to higher complexity levels. This transition is associated with new distinctive qualities and relationships, e.g. the “psyche” emerges from neuronal activity. In turn, these higher levels are superordinate to lower levels and set the boundary conditions for them (25). Based on this model recent studies as the paper by Hicks et al., evidence that psychiatric disorders can be mapped, represented and characterized (26). Recent theoretical developments suggest that quantum physics may add an additional conceptual dimension to this integrative framework (27). While not implying that the brain operates as a quantum computer, quantum theory introduces models of potentiality, complementarity, non-local correlations, and observer-dependent state collapse that resonate metaphorically and formally with nonlinear psychiatric dynamics. In particular, the notion that systems can exist in multiple potential states before “collapsing” into a measurable condition parallels the observation that human psychological states often fluctuate within fields of possibility before crystallizing into symptoms, decisions, or behaviors. Moreover, quantum-inspired approaches underscore the role of intentionality, attention, and relational context in shaping system trajectories—an idea consistent with therapeutic change processes and contemporary models of brain–mind interaction (27, 28).

When it comes to the second epistemological question—the relationship between brain and mind—it is noteworthy that Varela and Maturana proposed, from the 1980s onward, that neural activity runs in parallel with phenomenological domains, a position later described as neurophenomenology. From this perspective, psychology cannot be conceived as a mere extension of neuropsychology, but represents an autonomous scientific domain concerned with lived experience, meaning-making, and behavior as emergent phenomena situated at the interface between biological, relational, and cultural processes (10, 29).

While this perspective represented an important step beyond classical reductionism, it also entailed implicit forms of neurocentrism, namely the assumption that mental phenomena and their contents would ultimately be fully measurable in terms of brain-based processes alone (30).

In the present framework, measurable regulatory circuits are therefore not conceived as exerting a unidirectional influence on thoughts, emotions, behaviors, and biological rhythms; rather, their effects are dynamically reciprocal and co-determined by lived experience, interpersonal interaction, and socio-cultural contex (10, 29, 31). From an enactive and relational perspective, higher-order mental functions emerge within domains of interaction and intersubjective coordination, rather than being localized within the individual brain alone (32). Accordingly, while the brain can be regarded as a necessary condition for experiential life, it is not sufficient to account for subjectivity, agency, meaning, self-awareness, or higher-order cultural expressions such as art, religion, and science.

In response to the limitations of neurocentric paradigms, neo-existentialist philosophy has advanced a non-reductive conception of mind grounded in the continuous interplay between self, world, and other subjectivities (33). Within this view, mental phenomena—such as thoughts, perceptions, intentions, and the sense of self—are not mere by-products of neural activity, but dynamically evolving “self-portraits” through which the mind constitutes itself over time along a unique and subjective trajectory. Psychiatric symptoms, therefore, cannot be understood solely in terms of structural or functional neural alterations, but are shaped by the subjective and meaning-laden ways in which distress is experienced and expressed.

From the perspective of the proposed multi-level framework, meaning is not conceived as an additional static layer, but as a higher-order organizational domain that constrains and modulates processes across biological, psychological, and social levels. Within a systems and network perspective, existential meaning may be operationalized as a relatively stable configuration of narratives, values, expectations, and anticipatory models through which individuals interpret experience and regulate action over time. Formally, meaning functions as a top-down configurational pattern that shapes attractor stability, modulates coupling strength, and alters system sensitivity to perturbation across lower-level networks. In this sense, meaning does not reside in discrete representations but emerges as an integrative constraint influencing the dynamics of distributed regulatory processes. Existential crises can therefore be understood as phases in which these meaning-based constraints lose coherence or predictive adequacy, resulting in increased variability, loss of regulatory stability, and heightened vulnerability to maladaptive attractor states.

Synergetic theory provides a unifying framework for these considerations by describing how complex systems undergo critical phases in which small perturbations can generate new patterns of organization. Process-based psychotherapy operates precisely within such kairos moments—windows of heightened sensitivity during which transitions toward more adaptive attractor states become possible (34, 35). From this perspective, psychiatric care may be understood as a practice of restoring meaning and regulation, informed by multiple sources of information, including biological data, idiographic process mapping, and the patient’s lived context. If the mind is not reducible to the brain but instead constitutes a dynamic field of meaning, psychiatric illness can thus be conceptualized as a crisis in the processes that regulate this field.

To render these principles more concrete, the following section introduces four fully fictional clinical vignettes, designed solely as heuristic illustrations of the dynamics outlined above.

## Clinical vignettes illustrating a multilevel synergetic–neo-existential framework

### Case 1 – schizophrenia: transition into a new attractor state

L., a 23-year-old male university student, presented with a first-episode psychosis after several months of reduced social interaction, insomnia, and increasing preoccupation with existential themes.

**Figure 1:**
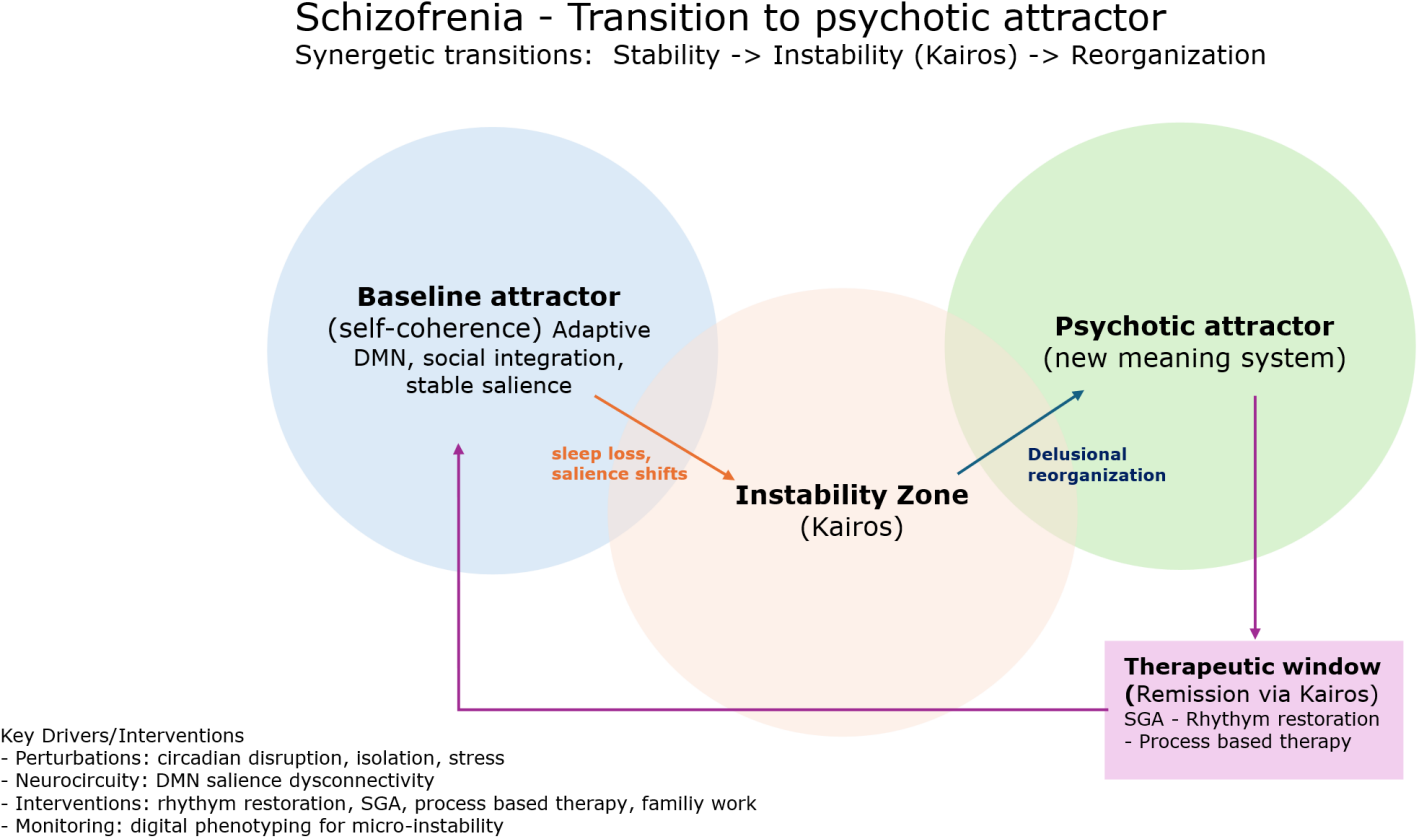
Transition into a psychotic attractor state. The attractor landscape is conceptually informed by neural mass and population-level dynamical models (e.g., Jansen–Rit and Wilson–Cowan), illustrating a kairotic transition into a locally stable but maladaptive psychotic regime following loss of system resilience.

From a dynamical systems and synergetic perspective, L.’s clinical course can be conceptualized as a transition between meaning-bearing attractor states (**Figure 1**). The prolonged phase characterized by insomnia, social withdrawal, and escalating existential preoccupation reflects a progressive destabilization of previously coherent meaning structures, accompanied by increased variability across circadian, affective, and relational regulatory loops. In complex systems terms, this loss of stability corresponds to a reduction in resilience and the emergence of early warning signals preceding a critical transition.

As the system approaches a critical threshold (kairos), meaning itself undergoes reorganization and begins to function as a maladaptive higher-order constraint. The psychotic state can thus be understood not merely as symptom accumulation, but as the establishment of a new psychotic attractor in which aberrant salience attribution and rigid self-referential interpretations provide a globally coherent—yet biologically, psychologically, and socially costly—configuration of meaning. In this sense, psychosis represents an internally stabilizing solution to a crisis of meaning, one that reduces uncertainty at the expense of flexibility, intersubjective validation, and long-term adaptability.

The kairotic phase denotes a temporally limited window during which this emerging attractor has not yet consolidated fully and remains sensitive to targeted perturbations. Multidisciplinary intervention during this phase does not aim at restoring a prior baseline state, but at facilitating the emergence of a new, more adaptive configuration of regulation and meaning. Biological stabilization (e.g., restoration of sleep–wake rhythms and modulation of dopaminergic salience), relational containment, and meaning-oriented psychotherapeutic work function as coordinated control parameters that reshape the landscape of possible attractors The therapeutic objective is thus the establishment of a novel, more resilient equilibrium—one that integrates subjective meaning with bodily regulation and social embeddedness.

Within this framework, digital phenotyping contributes to the empirical operationalization of kairos by enabling real-time detection of micro-instability patterns (e.g., fluctuations in activity–sleep rhythms), thereby supporting timely, individualized intervention before the psychotic meaning-attractor becomes rigidified.

### Case 2 – Major depression

Collapse Into a Low-Complexity Attractor A., a 46-year-old female nurse, developed severe depressive symptoms after prolonged occupational strain and the traumatic loss of a colleague.

**Figure 2:**
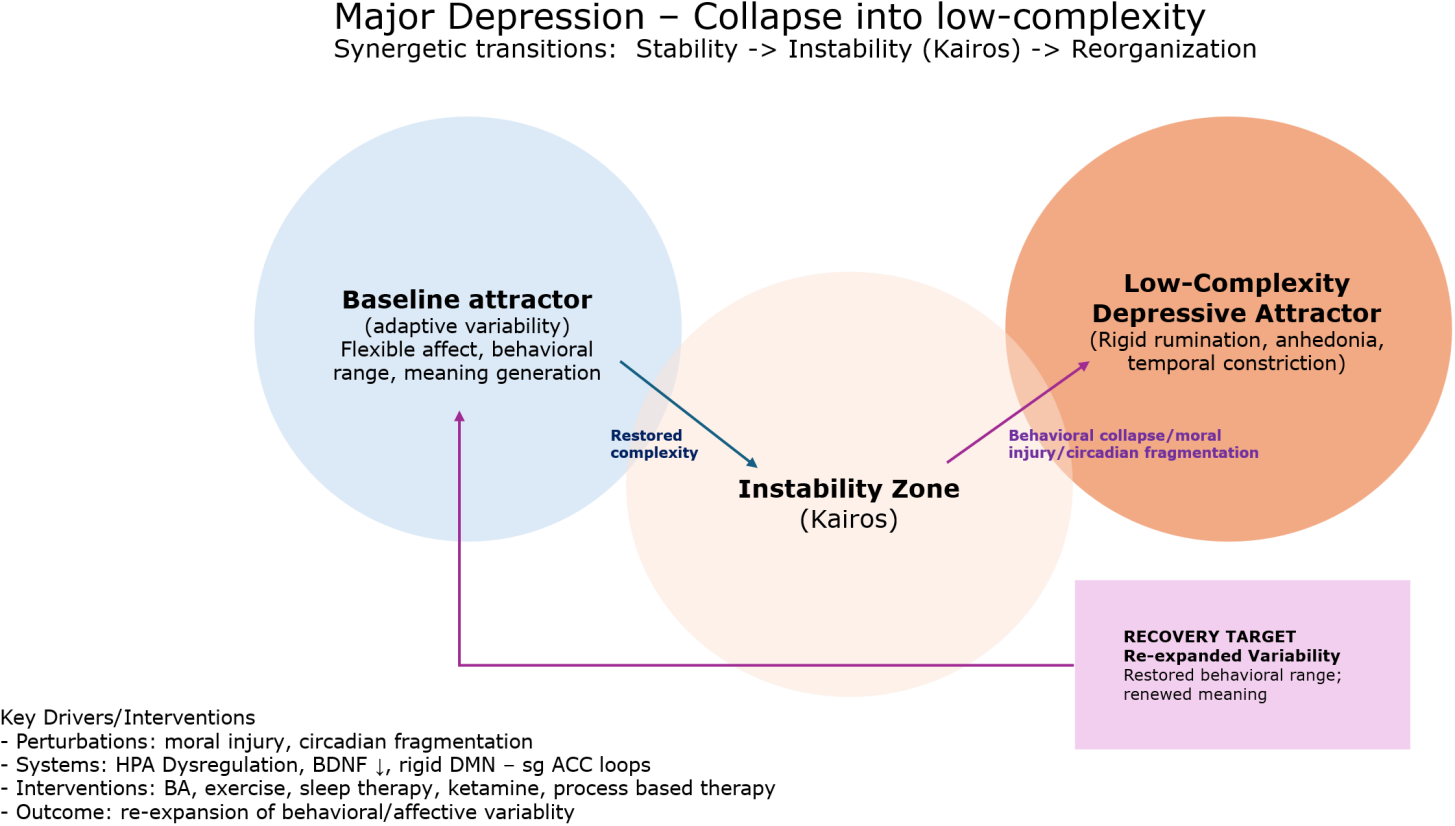
Collapse into a low-complexity depressive attractor. The schematic reflects nonlinear dynamical systems principles, depicting reduced variability and rigidity consistent with low-complexity attractor states described in neural population and dynamical models.

From a dynamical systems perspective, A.’s depressive episode can be conceptualized as a collapse into a low-complexity attractor characterized by reduced variability, diminished responsiveness to perturbation, and marked loss of adaptive degrees of freedom (**Figure 2**). Prolonged occupational strain and traumatic loss progressively constrained biological, psychological, and social regulatory loops, resulting in a rigid system state dominated by repetitive rumination and affective inertia. Such low-variability attractors are characteristic of systems that have lost the capacity for flexible reorganization despite ongoing environmental demands.

At the level of meaning, depression in this case reflects not merely an absence of significance, but the stabilization of a globally pessimistic and temporally constricted meaning configuration that functions as a higher-order constraint. Meaning becomes organized around themes of irreversibility, guilt, and hopeless anticipation, thereby reinforcing attractor rigidity and further reducing the system’s sensitivity to corrective input. In this sense, depressive suffering corresponds to a pathologically stabilized meaning attractor that minimizes uncertainty at the cost of vitality, openness, and future orientation.

Therapeutic intervention within this framework aims not at restoring a previous baseline, but at increasing system complexity and reopening trajectories of possible reorganization. Behavioral activation, exercise, and neuromodulatory interventions function as destabilizing control parameters that introduce variability into the system, while process-based psychotherapy and social repair processes target the depressive meaning configuration directly by reintroducing temporality, agency, and purpose. The therapeutic goal is the emergence of a new, more flexible equilibrium in which biological regulation, social connectedness, and meaning-making processes are mutually supportive rather than mutually constraining.

### Case 3 – Personality disorder: oscillatory attractor dynamics M., a 29

**Figure 3:**
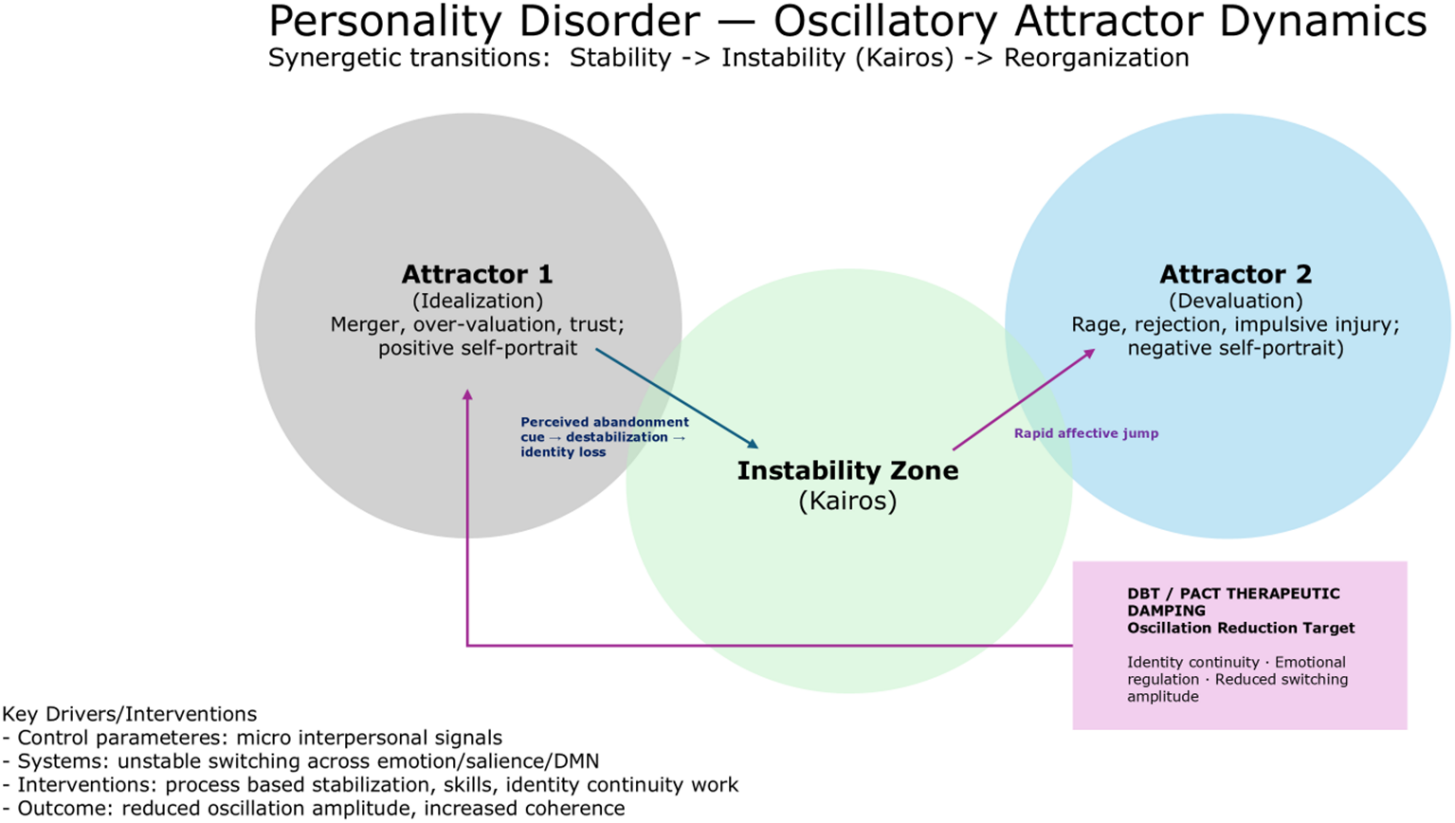
Oscillatory attractor dynamics in personality disorder. Rapid switching between shallow attractors is illustrated, conceptually grounded in nonlinear models of coupled dynamical systems and Wilson–Cowan–type frameworks sensitive to relational control parameters.

In dynamical systems terms, M.’s clinical presentation can be understood as a pattern of rapid switching between competing attractor states rather than entrapment within a single stable regime (**Figure 3**). The system displays high sensitivity to relational control parameters, such that minor interpersonal cues—particularly those signaling perceived abandonment—are sufficient to provoke abrupt phase transitions between affective and cognitive states. This form of oscillatory dynamics reflects insufficient damping and weak integrative coupling among regulatory loops, leading to chronic instability rather than rigidity.

At the level of meaning, this instability manifests as fragmented and short-lived self-configurations that fail to exert sustained top-down constraint on affect and behavior. Meaning does not stabilize into a coherent attractor but fluctuates rapidly with relational context, producing a succession of transient self-portraits organized around momentary emotional states. As a consequence, identity coherence remains fragile, and the system repeatedly reenters kairotic micro-windows characterized by heightened risk for impulsive acts or self-injury.

Therapeutic intervention, accordingly, does not primarily aim at destabilization or activation, but at increasing temporal continuity and attractor coherence. Process-based stabilization, dialectical-behavioral techniques, and identity-focused work function as regulatory mechanisms that enhance damping and strengthen higher-order meaning constraints. The clinical objective is the gradual emergence of meaning structures capable of maintaining continuity across affective fluctuations, thereby transforming chaotic attractor switching into more stable, resilient system dynamics. 

### Case 4 – ADHD (adult)

**Figure 4:**
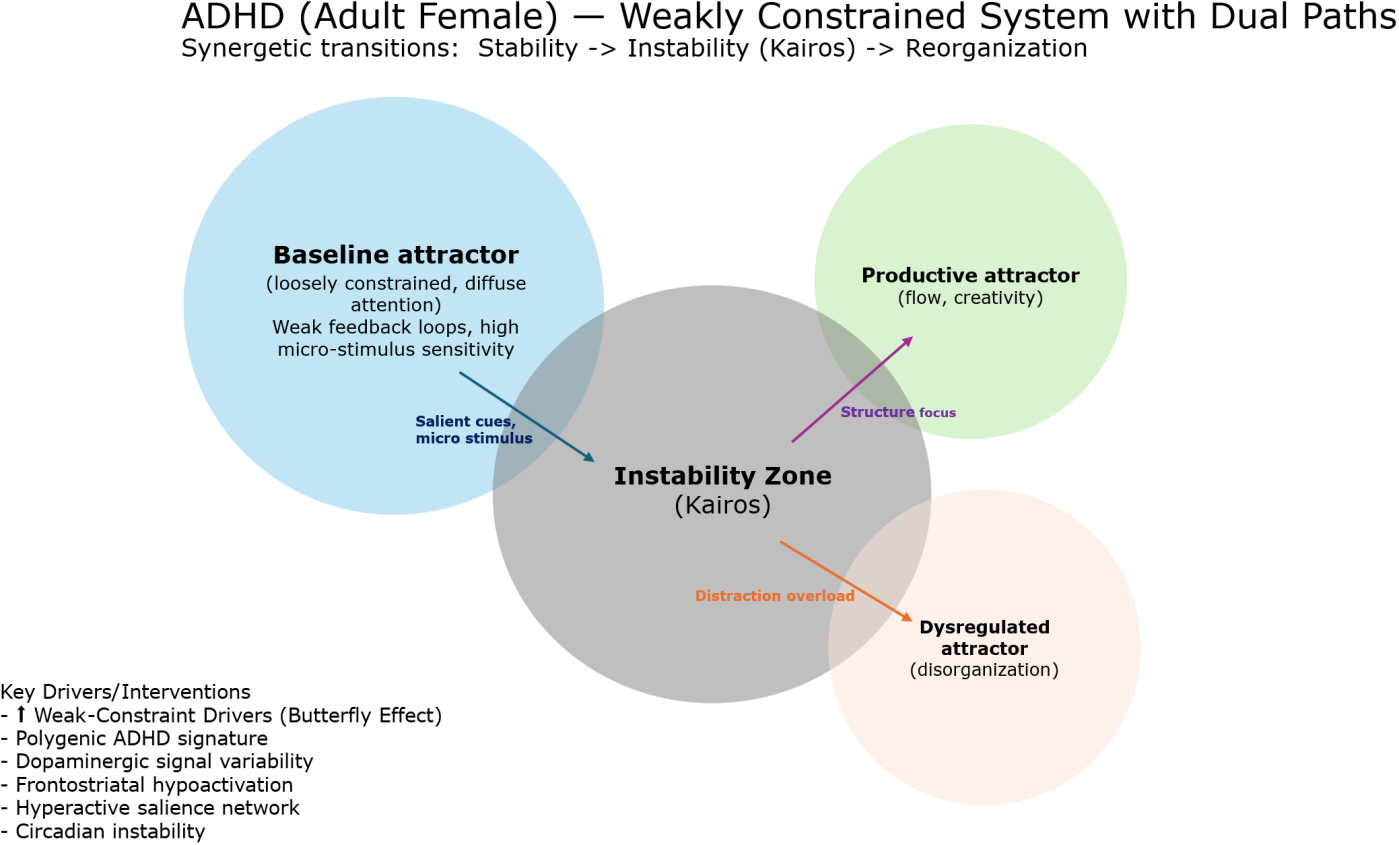
Weakly constrained regulatory dynamics in adult ADHD.

A Developmental Configuration of Weakly Constrained Regulatory Loops T., a 34-year-old woman working in graphic design, reported lifelong difficulties with attention, impulsivity, and organization, along with periods of intense creativity and hyperfocus (**Figure 4**).

From a synergetic perspective, T.’s ADHD presentation reflects a developmental configuration of weakly constrained regulatory loops, characterized by high sensitivity to minor perturbations and insufficient stabilization of attentional and executive control processes. Rather than converging toward a single dominant attractor, the system oscillates among multiple shallow attractor basins, allowing rapid shifts between distraction, impulsivity, and periods of hyperfocus. This configuration explains both functional impairments and context-dependent strengths, such as creative flow states.

At the level of meaning, these fluctuating dynamics give rise to markedly variable self-portraits that change across situational contexts (“incompetent,” “gifted,” “misunderstood”). Meaning therefore fails to operate as a stable higher-order constraint, resulting in inconsistent anticipatory models of the self and the future. The absence of a sufficiently stabilizing meaning attractor amplifies vulnerability to shame, external evaluation, and motivational collapse, particularly in structured social environments.

Therapeutic intervention within this model does not aim at rigidly constraining variability, but at shaping regulatory loops in a way that preserves adaptive flexibility while preventing dysregulation. Pharmacological treatment, sleep phase realignment, and process-based coaching function as modulatory control parameters that deepen adaptive attractors and increase predictability. Meaning-oriented identity work supports the integration of diverse self-experiences into a coherent narrative framework. The therapeutic goal is thus the establishment of a more robust, yet flexible, equilibrium that supports both functional regulation and creative potential. 

The attractor landscape is informed by dynamical systems models of frontostriatal control, illustrating shallow basins that permit frequent transitions between dysregulated and adaptive (e.g., flow) states.

Across all four cases, psychopathology emerges not as the loss of meaning, but as its pathological reconfiguration within maladaptive attractor states, underscoring that psychiatric treatment aims primarily at facilitating the emergence of new, more resilient equilibria rather than restoring a presumed baseline normality.

## Conclusion

The historical evolution of psychiatry—from Kraepelin’s entity-based nosology to contemporary network and systems science—reveals that mental suffering can no longer be understood as a set of discrete, linear, and static disease categories. Insights from genomics, epigenetics, and neurobiology depict a probabilistic and multifactorial landscape in which vulnerability emerges from the continuous interplay among genes, cells, neural circuits, psychological processes, and socio-cultural contexts. Within this framework, synergetics offers a powerful conceptual language to describe how human systems move through phases of stability, instability (kairos), and reorganization, generating new patterns of experience and behavior. The four clinical cases presented—emergent psychosis, major depression, borderline dynamics, and adult ADHD—make this perspective concrete and clinically visible. Each case demonstrates that psychopathological states are not fixed entities but dynamic forms of self-organization arising from multilevel instabilities. In schizophrenia, for instance, circadian disruption precipitated a transition toward a psychotic attractor; in depression, the system collapsed into a low-complexity regime; in personality disorder, rapid oscillations between attractors illustrated the sensitivity to interpersonal micro-perturbations; and in adult ADHD, weakly constrained feedback loops allowed transitions either toward dysregulation or toward creative, productive attractors. These examples show that contemporary clinical practice must learn to read phenomena not merely as symptoms, but as dynamic processes in ongoing reorganization.

In parallel, the anthropology of mental suffering and the neo-existentialist view remind us that no biological level—however sophisticated—can exhaust the human phenomenon. The mind is a field of meaning that continually produces its own self-portraits, and psychiatric crises represent ruptures in the continuity between the subject and their world. Care, therefore, is not simply a matter of correcting dysfunction but of accompanying the person through transitions toward new, more coherent attractors of significance. The evolution of psychotherapies toward process-based, idiographic, and context-sensitive approaches fits precisely within this conceptual horizon. Within this multilayered and dynamic scenario, Artificial Intelligence is emerging as a transformative companion. Predictive models, digital phenotyping, and nonlinear analytical tools make it possible to detect micro-instabilities, patterns of variability, critical windows of change, and early signals of transition (36–38). AI does not replace clinical judgment; it augments it. It provides dynamic maps of individual systems, supports diagnosis based on evolving clusters rather than static categories, guides adaptive therapeutic choices, and contributes to the development of more integrated and responsive mental-health ecosystems. An important implication of the present framework concerns future clinical-operational work. While the schematic vignettes presented here effectively illustrate distinct dynamic patterns across levels, an idiographic and longitudinal single-case investigation would be better suited to capture the temporal depth of kairotic transitions and related clinical decision-making processes. In-depth single-case studies therefore represent a natural extension of the current model and a key direction for future research.

Looking ahead, psychiatry is called to become part of a genuine ecology of mental health, in which biological, psychological, social, technological, and existential levels interact continuously. The framework proposed in this work—rooted in complexity theory, an anthropology of the self, and rigorous ethical responsibility—is not merely a conceptual synthesis, but a practical orientation for clinical care.

Within this perspective, digital tools and artificial intelligence are conceived as supportive and assistive resources, not as substitutes for human judgment, relational understanding, or clinical responsibility. Their use presupposes transparency, informed consent, and the preservation of patient autonomy, ensuring that technological monitoring enhances rather than constrains personal agency and lived experience.

Understanding psychiatric disorders as transitions among attractor states; intervening through process-based therapies during kairotic windows; employing AI cautiously as an augmentative—rather than directive—instrument; and recognizing subjectivity as an embodied field of meaning together delineate a possible “new era” of psychiatry. In this view, the task of clinical practice is no longer to restore a lost normality, but to accompany human systems in their evolution toward configurations that are more coherent, flexible, and meaningful. The psychiatry of the future will thus be not only more technological, but also more human—more systemic, more ethical, and more capable of generating understanding, relationship, and possibility.

## Data Availability

The original contributions presented in the study are included in the article/supplementary material. Further inquiries can be directed to the corresponding author.
